# A New Class of High‐Capacity Fe‐Based Cation‐Disordered Oxide for Li‐Ion Batteries: Li‐Fe‐Ti‐Mo Oxide

**DOI:** 10.1002/advs.202300615

**Published:** 2023-04-23

**Authors:** Jieun Kim, Yongho Shin, Byoungwoo Kang

**Affiliations:** ^1^ Department of Materials Science and Engineering Pohang University of Science and Technology (POSTECH) 77 Cheongam‐Ro, Nam‐Gu Pohang 37673 Republic of Korea; ^2^ Research Institute of Industrial Science and Technology (RIST) POSCO Global R&D Center 100 Songdogwahak‐ro Yeonsu‐gu Incheon 21985 Republic of Korea

**Keywords:** cation disordered rocksalt lithium excess materials, Fe‐based electrode materials, high capacity materials, lithium ion batteries

## Abstract

Low‐cost Fe can be used for forming cation‐disordered rocksalt Li‐excess (DRX) materials instead of high‐cost d^0^‐species and then the Fe‐based DRX can be promising electrode materials because they can theoretically achieve high capacity, resulting from additional oxygen redox reaction and stable cation‐disordered structure. However, Fe‐based DRX materials suffer from large voltage hysteresis, low electrochemical activity, and poor cyclability, so it is highly challenging to utilize them as practical electrode materials for a cell. Here, novel high‐capacity Li‐Fe‐Ti‐Mo electrode materials (LFTMO) with high average discharge voltage and reasonable stability are reported. The effect of Ti/Mo on electrochemical reactions in Fe‐based DRX materials (LFTMO) is studied by controlling its composition ratio and using techniques for analyzing the local environment to find the key factors that improve its activity. It is found out that the introduction of appropriate quantity of redox‐active Mo^4+/5+^ to Fe‐based DRX materials can help stabilize the oxygen redox reaction via changing a local structure and can suppress a Fe redox reaction, which can cause poor performance. The understandings will help develop high capacity and long cyclability Fe‐based DRX electrode materials.

## Graphical abstract

Novel high‐capacity Li‐Fe‐Ti‐Mo electrode materials (LFTMO) with high average discharge voltage and reasonable stability are reported. Introducing appropriate amount of redox‐active Mo^4+/5+^ can change the local structure and limit the Fe‐related redox reaction, resulting in improving the reversibility of the oxygen redox. This understanding enables designing the high‐capacity and long cyclability Fe‐based disordered rocksalt electrode materials.

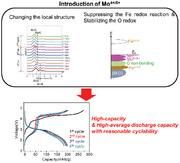

## 1 Introduction

In Li‐ion batteries (LIBs), the development of high‐capacity electrode materials is required to meet the growing demand for not only powering the state‐of‐art portable electronic devices but also providing large‐scale applications such as electric vehicles and grid‐scale energy storage system.^[^ ^1^ ^]^ Especially, the achievable capacity of cathode materials is considered to be the bottleneck for achieving high energy density of LIBs.^[^ ^2^ ^]^ In this regard, Li‐rich oxide materials have been intensely studied as a new paradigm to increase the energy density of electrode materials because they can achieve much higher specific capacity (>300 mAh g^‐1^) than traditional layered cathodes such as LiCoO_2_ and LiNi*_x_* Co*_y_* Mn_1‐_ *_x_*_‐_ *_y_* O_2_ (NCM) via using both the anion and cation redox reaction.^[^ ^3^ ^]^ Oxygen redox reaction in Li‐excess materials mainly occurs by extracting labile electrons from unhybridized O 2p states created by Li‐O‐Li configurations.^[^ ^4^ ^]^ Among Li‐rich oxide materials, cation‐disordered rocksalt Li‐excess (DRX) materials, where both Li and transition metal (TM) distribute randomly in octahedral sites in a layered structure, have received lots of attention owing to their high capacity and stability.^[^ ^5^ ^]^ Li‐O‐Li configurations in DRX materials can be more than those in the typical Li‐excess layered materials even with the same excess Li amount. Furthermore, the DRX materials can have advantages over the general layered materials because the cation‐disordered structure is stable even when a significant amount of Li is extracted due to the weak repulsive force between layers and its high entropy, which results in the minimal structural change upon cycling.^[^ ^6^ ^]^

Fe is the most abundant element in the Earth crust, and more environmentally benign than Co.^[^ ^7^ ^]^ To synthesize the DRX materials, large amount of high‐cost 4d and 5d d^0^ or d^10^ species is typically needed because d^0^ or d^10^ species can enable to easily accommodate local strains and a facile disorder leading to the formation of the cation‐disordered structure.^[^ ^8^ ^]^ Although the low‐cost 3d‐transition metal Fe^3+^ ion is not a d^0^‐TM, it can be used as a substitute for d^0^ or d^10^‐species to induce the cation‐disordered structure because it easily induces randomly mixing of Li due to its similar ionic size with Li^+^ ion and its charge.^[^ ^9^ ^]^ Unfortunately, Fe‐based DRX materials suffer from large voltage hysteresis, low reversibility, and low reversible activity, making it difficult to use practically.^[^ ^9^, ^10^ ^]^ Moreover, particles in typical Fe‐containing DRX material can be easily pulverized during charge and discharge process, resulting from the Fe redox reaction or oxygen redox reaction and structural reconstruction process.^[^ ^11^ ^]^ Therefore, it is important to understand the reaction mechanism and develop high‐performance and stable Fe‐based DRX materials while minimizing the amount of expensive 4d and 5d d^0^ species.

Since the main redox active element in DRX materials is not a d^0^‐TM, which can typically maintain the cation‐disordered structure during cycling, electrochemical activity in the DRX materials is mainly from non‐d^0^‐TM elements, which can activate cationic or/and anionic redox reaction to achieve high capacity. Many DRX materials have Mn or Ni^[^ ^8a^ ^]^ as active redox elements because they are operated well in the cation‐disordered structure. There are several Fe‐containing DRX materials that can have poor electrochemical activity with limited reversibility.^[^ ^12^ ^]^ For example, Li‐Fe‐Ti‐O based DRX materials have been reported to optimize electrochemical performance by controlling the ratio of Li to TM, compositional control rather than understanding the charge/discharge mechanism has been focused on.^[^ ^13^ ^]^ Most of Fe containing DRX shows a Fe redox activity as a transition metal redox reaction while the oxygen redox reaction is active. The use of Fe redox reaction can negatively affect entire electrochemical reaction and reversibility of Fe‐based DRX materials. Recently, a paper reported that when Fe^3+^/Fe^4+^ redox reaction is active in Li‐Fe‐Ti‐O, the charge transfer from O(2p) lone pair states to Fe(3d) states is involved with sluggish structural distortion and thereby the Li‐Fe‐Ti‐O can show large voltage hysteresis.^[^ ^10^ ^]^ Therefore, it is necessary to understand the redox reaction in Fe‐based DRX materials, which is not well understood in typical Fe‐based DRX materials.

In this study, we report on a novel cation‐disordered rocksalt‐type Li_1.2_Fe_1/3_Ti*_x_*_/15_Mo_(7‐_ *_x_*_)/15_O_2_ (*x* = 3, 4, 5, and 6, denoted as LFTMOs hereafter) to understand the relationship between the Ti/Mo ratio and electrochemical properties. A series of LFTMOs have been synthesized via a solid‐state reaction, followed by carbon coating process. The structure, electrochemical properties, and redox reaction mechanisms of the as‐prepared LFTMOs were probed by using techniques including X‐ray diffraction and X‐ray absorption spectroscopy (XAS). Compared with other Fe‐containing DRX materials,^[^ ^13^ ^]^ the sample with *x* = 4 that has a certain amount of Mo shows high average discharge potential (≈2.76 V) and high capacity (≈237.9 mAh g^‐1^) even at room temperature. The high average discharge voltage can be partly ascribed to the fact that the Fe redox reaction in LFTMOs is not activated while mainly preserving the disordered structure. This is a quite different behavior from the previous reports that Fe generally participates in redox reaction, leading to the discharge voltage decay and structural instability.^[^ ^11^ ^]^ During the first charge process, local structure of Mo in LFTMOs is modified and then maintained in subsequent cycles. The modified local structure of Mo can not only activate its electrochemical reaction but also stabilize the oxygen redox reaction. As a result, the LFTMO sample can achieve high reversible capacity even with Fe. Even if LFTMO is also compared to the commercial NCM, LFTMO can have a higher capacity due to additional oxygen redox reaction and cost advantage because LFTMO contains Fe and Ti instead of Co. We believe the understanding and findings will pave the way to develop a low‐cost and high‐energy‐density Fe‐based DRX materials.

## 2 Results

### 2.1 Structural Characterizations of Li_1.2_Fe_1/3_Ti*_x_*_/15_Mo_(7‐_ *_x_*_)/15_O_2_

**Figure 1:**
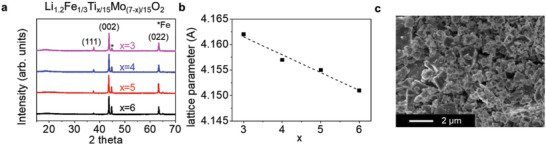
Structural characterization of LFTMO samples with different amount of Ti and Mo. a) X‐ray diffraction patterns. b) The change in lattice parameters of the samples. c) SEM image of the *x* = 5 sample.

Li_1.2_Fe_1/3_Ti*_x_*_/15_Mo_(7‐_ *_x_*_)/15_O_2_ (*x* = 3, 4, 5, 6) (denoted as LFTMOs hereafter) were synthesized through a simple solid‐state reaction, followed by a carbon coating (Details in Experimental Section). The structure of the LFTMOs, which are carbon‐coated samples, was determined by X‐ray diffraction (XRD). The absence of (003) peaks near 18°, which is the main and characteristic peak of the typical layered oxide materials, in the LFTMOs indicates that the cation ordered layered structure was not synthesized. The LFTMOs have a cation‐disordered rocksalt structure (*α*‐LiFeO_2_) with $Fm\bar{3}m$ space group, wherein all the cations randomly distribute in the octahedral 4a site (**Figure** **1**a and Figure S1, Supporting Information). As a result, the LFTMOs were considered as a cation‐disordered rocksalt Li‐excess (DRX) material. Also, all samples have certain amount of a Fe metal, which is indexed to bcc Fe ($Im\bar{3}m$ space group), as an impurity minority phase. The Fe metal can be caused by the reduction process at high temperature.^[^ ^14^ ^]^ Details are listed in Table S1 (Supporting Information). The cation disordered rocksalt phase and Fe metal impurity phase were also detected in synthesized samples without the carbon coating (Figure S2, Supporting Information). This indicates that the carbon coating could be not the only cause of the Fe metal impurity. As a result, according to XRD data, the disordered rocksalt phase in the LFTMOs has less amount of Fe than the intended amount Fe due to the existence of Fe metal as an impurity phase. Actual composition was calculated based on the amount of Fe metal impurity/DRX phase from refined XRD results (Table S2, Supporting Information)

The lattice parameters were obtained from Rietveld refinements (Figure 1b). The lattice parameters of the LFTMOs linearly decrease from 4.162 Å (*x* = 3) to 4.151 Å (*x* = 6) according to the increase in the amount of Ti (the decrease in the amount of Mo). This indicates that the samples can follow Vegard's law.^[^ ^15^ ^]^ This can be ascribed to the doping of Ti^4+^ (0.605 Å) into the structure, which has a smaller ionic size than Mo^4+^ (0.65 Å) and Mo^5+^ (0.61 Å). As a result, the Ti instead of Mo can be doped into the bulk of the samples. Typical particles of the LFTMOs (the sample with *x* = 5) show wide range of particle size distribution from several hundred nm to about 2 µm, and the particles show an irregular faceted shape (Figure 1c). Also, transmission electron microscopy (TEM) and electron energy loss spectroscopy (EELS) clearly show homogeneous element mapping of the Li, Fe, Mo, Ti, and O in a particle in the sample with *x* = 5 (Figure S3, Supporting Information). This indicates that the Mo and Ti could be incorporated into the bulk structure in the LFTMOs.

### 2.2 Electrochemical Properties of the LFTMO Samples

**Figure 2:**
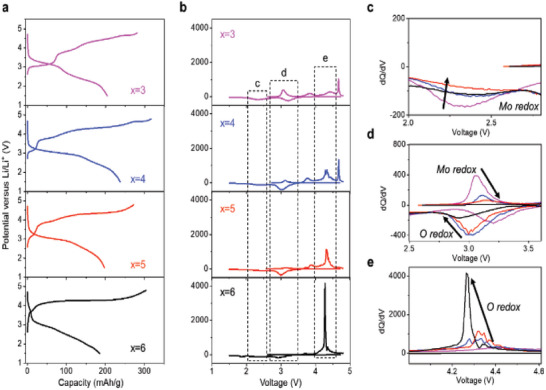
Electrochemical properties of the LFTMOs. a) Voltage profiles and b) differential capacity plots (d*Q*/d*V*) of LFTMOs during first cycle. (Current density: 5 mA g^‐1^ and cutoff voltage from 1.5 to 4.8 V). c–e) d*Q*/d*V* curves of enlarged regions of (b).

The electrochemical properties of the LFTMOs samples were evaluated with a constant current density of 5 mA g^‐1^ with cutoff voltage range from 1.5 to 4.8 V (vs Li/Li^+^) at room temperature (**Figure** **2**a). Among the samples, the sample with *x* = 4 shows highest charge capacity, ≈317 mAh g^‐1^, and highest discharge capacity, ≈238 mAh g^‐1^ leading to high coulombic efficiency, ≈75%. The charge and discharge capacity and coulombic efficiency of each sample are summarized in Table S3 (Supporting Information). The samples show quite different charge/discharge curve at first cycle indicating that the electrochemical reactions are different in the samples. Their electrochemical behavior of the samples during the first charge process involves a reaction near 3 V (Figure 2d), which can be related to a transition metal redox reaction, and a plateau reaction above 4.2 V (Figure 2e), which can be ascribed to the oxygen redox reaction as reported from previous study.^[^ ^10^ ^]^ As the Ti amount (*x*) increases and Mo amount decreases, the redox reaction at ≈3 V in the charge process becomes shorter and finally almost disappears. This indirectly indicates that the redox reaction at ≈3 V in the first charge can be related to the Mo redox reaction. The reaction near 3.8 V in the charge may be related to the additional Mo redox reaction that can be due to a structural rearrangement that usually occurs in Li_2_MoO_3_,^[^ ^16^ ^]^ whereas the reaction near 4.7 V in the charge of *x* = 3 and 4 samples is likely caused by electrolyte oxidation. This is consistent with the reported molybdenum redox reactions in the Li_2_MoO_3_‐based materials previously^[^ ^17^ ^]^ and also will be discussed in detail later. The samples during the first discharge process have two redox reactions around ≈3 V and below 2.5 V. The redox reaction at ≈3.0 V (Figure 2d) can be attributed to the oxygen redox reaction^[^ ^18^ ^]^ and the other reaction below 2.5 V (Figure 2e) can be related to Mo redox reaction.^[^ ^8a^ ^]^ This indicates that the redox reactions are partially reversible in the samples. All reactions are clearly shown on the differential capacity plot with respect to voltage (d*Q*/d*V* plot) (Figure 2b–e), in which the peaks correspond to the reactions in Figure 2a.

As the Ti amount (*x*) increases from *x* = 3 to 6 and the Mo amount decreases (Figure 2d,e), the length of plateau above 4.2 V, which is related to the oxygen redox reaction, during first charge increases at the expense of ≈3 V reaction, leading to a flatter profile. This result is consistent with the reaction in Li_2_TiO_3_‐based Li excess materials.^[^ ^19^ ^]^ However, during the discharge, the oxygen redox reaction decreases as the Ti amount increases except the sample with *x* = 3, which barely shows the oxygen redox reaction upon charge. On the basis of this, we predict as Ti amount (*x*) increases, the reversibility of the oxygen redox reaction can be worsened, whereas as Mo increases, an additional molybdenum redox reaction occurs and simultaneously the reversibility of oxygen redox reaction can be increased. As a result, it was found that introduction of Mo is conducive to enhancing the reversibility of the oxygen redox and inducing the increase in the Mo redox reaction in the LFTMO samples. These reactions were optimized at the sample with *x* = 4 with respect to optimal molybdenum redox reaction and oxygen redox reaction, resulting in the highest discharge capacity (237.9 mAh g^‐1^) with high discharge voltage among the samples. The sample with *x* = 4 also had a higher discharge capacity than those in the reported papers (Table S4, Supporting Information).

### 2.3 Understanding Electrochemical Redox Reactions by Using X‐Ray Absorption Near Edge Spectroscopy (XANES)

To understand the redox reactions in the samples, the representative sample with *x* = 5 was studied in detail. The atomic compositions of the sample with *x* = 5 were measured by inductively coupled plasma (ICP) (Table S5, Supporting Information). The measurements were almost identical to the target nominal composition. To determine the oxidation state of the sample with *x* = 5, X‐ray absorption near‐edge spectroscopy (XANES) measurements were carried out before cycle. The Fe K‐edge and Ti K‐edge XANES spectra of the pristine sample with *x* = 5 (Figure S4a,b, Supporting Information) show that the iron and titanium oxidation states of the sample are Fe^3+^ and Ti^4+^, respectively. The facts that the edge spectra of Mo in the sample with *x* = 5 is located between Mo^4+^ in MoO_2_ and Mo^6+^ in MoO_3_ and pre‐edge peak of Mo, which can be clearly observed in Mo^6+^ in MoO_3_ caused by the octahedral distortion, is negligibly observed, suggest that Mo has an oxidation states between 4+ and 5+ (Figure S4c, Supporting Information).^[^ ^20^ ^]^ The targeted oxidation states were Fe^2+^, Ti^4+^, Mo^6+^, but the oxidation states of transition metal ions were modified partly because some Fe ions were reduced into Fe metal and Fe was stabilized with 3+. As a result, the Mo can have lower oxidation state than the targeted oxidation state, 6+.

**Figure 3:**
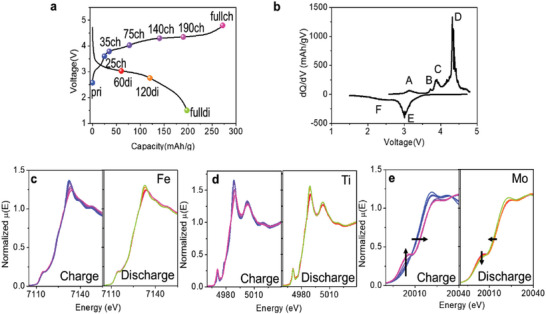
Redox mechanism of the sample with *x* = 5. a) Voltage curve for first cycle and b) d*Q*/d*V* curve in the voltage range 1.5–4.8 V at room temperature, (Current density: 5 mA g^‐1^). XANES spectra of c) Fe K‐edge, d) Ti K‐edge, and e) Mo K‐edge during first cycle.

The charge and discharge curves of the sample with *x* = 5 in the voltage range of 1.5–4.8 V at a current density of 5 mA g^‐1^ are shown in **Figure** **3**a. In the first cycle, the sample with *x* = 5 delivers charge capacity of 272.2 mAh g^‐1^ and discharge capacity of 197.4 mAh g^‐1^, leading to 72.7% coulombic efficiency. Figure 3b presents differential capacity plots with respect to voltage (d*Q*/d*V*) from the voltage profile in Figure 3a. In the first charge process, a high oxidation peaks D and additional small peaks A, B and C were observed. However, the discharge process observed only two reduction peaks: the peak E and a broad peak F.

The changes in the oxidation states of transition metals during charge and discharge were measured by the ex situ XANES measurements. Figure 3c–e and Figure S5 (Supporting Information) show Fe K‐edge, Ti K‐edge, and Mo K‐edge of the sample with *x* = 5 during first charge/discharge. It is also clear that the Fe ions in LFTMOs do not participate in the redox reaction because there is no edge shift, indicating there is no change in oxidation state of Fe ions during charge and discharge. Also, Ti^4+^ remains inactive during charge/discharge because Ti^4+^ cannot be oxidized beyond the tetravalent limit. This indicates that neither Fe nor Ti participates in electrochemical redox reaction in the sample. Interestingly, Mo K‐edge peak shows a large shift unlike Fe and Ti K‐edge peak, demonstrating that among the transition metals, only Mo ions participate in the redox reaction during charge and discharge. This indicates that the transition metal redox reaction in first charge process is related to the Mo, not Fe.

Specifically, at charge, Mo K‐edge peak shows a large shift toward higher energy and the increase in pre‐edge intensity after ≈75 mAh g^‐1^. The increase in the pre‐edge intensity indicates the change in the oxidation state of Mo from 4+ ≈ 5+ to 6+ because the existence of the pre‐edge peak is a distinct characteristic of the distortion of Mo^6+^‐O_6_ octahedra.^[^ ^21^ ^]^ However, in 140 mAh g^‐1^ of the charge, the Mo oxidation state is almost saturated to 6+, and after then does not change any more. This indicates that the further oxidation is not from Mo redox reaction. At discharge, the edge in Mo K‐edge did not shift until ≈120 mAh g^‐1^ and after then its edge shifted to lower energy at the end of discharge, indicating that there was no change in oxidation state of Mo at the beginning of discharge until the 120 mAh g^‐1^ of discharge capacity was achieved, and then Mo^6+^ was reduced at the end of discharge. However, the oxidation state of Mo does not completely return to the pristine state, 4+ ≈ 5+. The pre‐edge peak in Mo K‐edge still remains even after the end of the discharge. This indicates that the redox reaction of Mo can be changed in subsequent cycles and local environment of Mo‐O_6_ has been distorted during first cycle. The change in the local environments of Mo can be related to the migration of cations in first cycle. Note that the Mo^4+^/Mo^6+^ redox reactions in the sample with *x* = 5 (Li_1.26_Fe_0.25_Ti_0.35_Mo_0.14_O_2_) can theoretically contribute a capacity of 88 mAh g^‐1^, whereas actual charge and discharge capacity of 272.2 and 197.4 mAh g^‐1^ were achieved, respectively. Thus, the extra capacity could originate from the oxygen redox reaction. Therefore, during first charge, Mo (A, B, and C) and O (D) are sequentially oxidized, and during discharge, O (E) is reduced at the beginning of the discharge and then Mo (F) is reduced at the end of discharge. According to the XANES results, Mo is oxidized during the beginning of charge and reduced at the end of discharge while the oxygen redox reaction occurs at the end of charge and at the beginning of the discharge. This indicates that the redox reactions of the LFTMO sample happen in the order: (charge) TM‐oxygen‐(discharge) TM‐oxygen redox reaction. This is quite different from typical Li‐rich layered oxide materials that have redox inversion behavior, (charge) TM‐oxygen‐(discharge)‐TM‐oxygen.^[^ ^22^ ^]^

In the second cycle (Figure S6, Supporting Information), the redox reaction of Mo is still contributed to the obtained capacity but the oxidation state of Mo does not return to the pristine state (4+ ≈ 5+). This indicates that the oxidation state and local structure of Mo are changed after the first cycle, and then the changed structures and the redox reaction are maintained in subsequent cycles. Furthermore, Fe and Ti in second cycle do not participate in the redox reaction. Given that the obtained capacity is much higher than the theoretical capacity of Mo redox reaction, ≈88 mAh g^‐1^, in the second cycle, the oxygen redox reaction is still activated to make a large contribution to the obtained capacity. This indicates that the partial Mo redox reaction and the oxygen redox reaction are reversible in the LFTMO samples. Compared to the previously reported Li‐Fe‐Ti‐O cathode and other general conversion‐type materials, where Fe ions participate in the redox reaction,^[^ ^23^ ^]^ the LFTMO sample has higher average discharge potential due to the absence of the Fe redox reaction, which typically occurs at low voltage, and higher capacity due to reversible oxygen redox reaction and the participation in redox reaction of Mo. Therefore, the incorporation of Mo can not only enhance the reversibility of the oxygen redox reaction but also increase the operating voltage in Fe‐containing DRX materials leading to high energy density.

### 2.4 Structural Evolution of the LFTMO Sample during Delithiation/Lithiation

**Figure 4:**
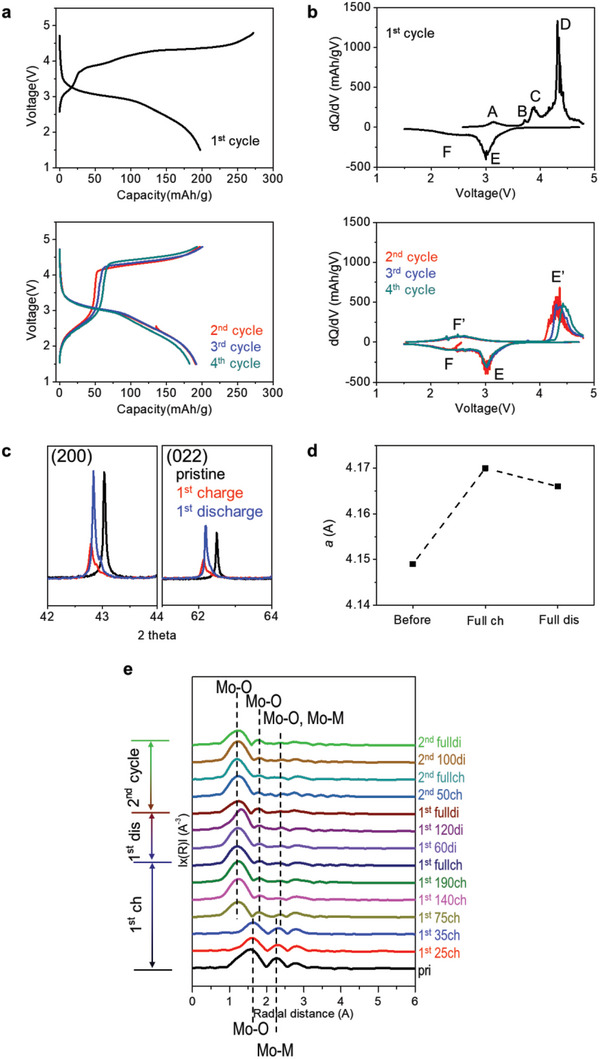
Electrochemical reversibility and involved structural changes during cycles. a) Voltage profiles of the sample with *x* = 5 in the voltage range 1.5–4.8 V at RT (current density: 5 mA g^‐1^). b) Differential capacity plots, d*Q*/d*V* obtained from voltage profiles in (a). c) Synchrotron X‐ray diffraction patterns of the sample with *x* = 5 during the first cycle at 5 mA g^‐1^ and d) the refined *a*‐lattice parameter (Full ch: fully charged state; Full dis: fully discharged state). e) Ex situ FT‐EXAFS spectra of the electrode of the sample with *x* = 5 during 2 cycles.

Electrochemical tests of the sample with *x* = 5 in subsequent cycles were performed to understand the electrochemical reversibility and structural changes (**Figure** **4**a). First of all, after the first cycle, the sample with *x* = 5 in Figure 4a was reversibly operated for cycles. The charge redox reactions in the subsequent cycles are different from those in first cycle. The redox peaks at A, B, and C in the first charge almost disappear in the charge of the subsequent cycles in Figure 4b. Unlike the redox reactions at first charge, new two distinct charge redox reactions below 3 V (F’) and above 4 V (E’) are observed in the subsequent cycles. In contrast, the discharge reactions in the first cycle are almost maintained in subsequent cycles (Figure 4b). During charging in the subsequent cycles, reactions at F’ and E’ can be related to Mo redox reaction and oxygen redox reaction, respectively (Figure S6d, Supporting Information). Therefore, reactions at E and E’, F and F’ can be coupled, respectively. This result demonstrates that the redox reactions are changed after first cycle and it can be related to the structural change caused by Mo redox reaction. Especially, this indicates that the Mo redox reaction during charge still occurs in the subsequent cycles but it is not as much as that in the first cycle, and the oxygen redox reaction remains continuously. Furthermore, the flat voltage of the oxygen redox reaction in the charge maintains in subsequent cycles. This unusual phenomenon could be probably the result of the change in local structure of Mo after first charge, which can change the electronic structure in subsequent cycles.

Figure 4c,d shows that the sample undergoes an irreversible structural change during first cycle. The (002) and (022) peaks were shifted to a lower angle after first charge, indicating the increase in cubic lattice parameter. This can be related to the change in the oxidation state of Mo to 6+ during first charge. The peak position of fully discharged sample did not return to the pristine structure. This indicates that the structural change irreversibly occurs during first cycle. Furthermore, the peak position of the discharged electrode barely changes compared to that of the charged electrode even though the intensity and FWHM (Full width at half maximum) of the peaks change after first discharge. Given that a shift in peak positions is typically observed in XRD results during delithiation and lithiation partly due to the volumetric change,^[^ ^6^ ^]^ negligible changes in XRD peak positions in LFTMO indicates that the changed structure of LFTMO can maintain stably without changing lattice parameter. After first discharge, the overall intensity of the peaks and their FWHM were also fully recovered even though the peak position was not recovered to the pristine. This indicates that the crystallinity of the sample with *x* = 5 is almost fully recovered unlike typical Fe‐containing cation disordered Li‐excess (DRX) materials that can undergo the pulverization of particle during cycling and in the end become amorphous state after just one cycle, resulting in low crystallinity.^[^ ^11^, ^24^ ^]^ SEM measurements before/after 50 cycles of the sample with *x* = 4 in Figure S7 (Supporting Information) further demonstrate that the particles do not have any severe pulverization and cathode electrolyte interphase (CEI) layer on the surface that is caused by the side reactions with the electrolyte.^[^ ^25^ ^]^

To understand the local structure surrounding Mo atom during charge/discharge, the Mo K‐edge extended X‐ray absorption fine structure (EXAFS) was measured. Figure 4e shows the Fourier transform of the *k* ^2^‐weighted EXAFS of Mo during first and second cycle. The first peak at ≈1.6 Å is related to Mo—O bond and the second peak at ≈2.2 Å is related to Mo—metal bond.^[^ ^20b^ ^]^ The first peak is not changed until the charge capacity was 75 mAh g^‐1^ in first charge. At 75 mAh g^‐1^ of charge capacity, this peak was split to the low radial distance. After the capacity of 75 mAh g^‐1^ at initial charge, the local environment of Mo—O bond barely change in subsequent cycles. Given that the redox reaction of Mo occurs in Figures 3 and 4, this movement can be related to the oxidation of Mo to 6+ because the oxidation state of Mo^6+^ easily induces the distortion of the octahedral environments and local migration.^[^ ^20b^ ^]^ During first charge, the lattice parameter can be changed because of local structural change during the first charge. These results demonstrate that during the first charge process, Mo can increase local disordering via the structural migration and thereby this can enable the redox reaction of Mo in subsequent cycles. Also, this local structural change can have positive effects on the oxygen redox reaction by stabilizing the structure and O state.^[^ ^26^ ^]^

## 3 Discussion

To understand the electrochemical redox reactions in the Fe‐based DRX materials, the reaction mechanism was studied by tuning the ratio of Ti/Mo. As the amount of Ti increased, the activity of the oxygen redox reaction increased but the TM redox reaction decreased during charging but the reversibility of the oxygen redox reaction decreased during discharging. In contrast, as the amount of the Mo increased, the reversible oxygen redox reaction increased and simultaneously Mo‐related transition metal redox reaction increased leading to high capacity. Even though the LFTMO samples have a certain amount of the Fe, Fe redox reaction barely occurs leading to suppression of lowering the discharge voltage. Among the samples, the sample with *x* = 4 can have the highest discharge capacity, 238 mAh g^‐1^ with high average discharge voltage via optimal oxygen redox reaction and Mo redox reaction.

The reason why the redox behavior changes depending on the ratio of Ti to Mo can be explained from the point of view of the electronic structure, which can be described to the relative values of the Mott‐Hubbard (*U*) and charge‐transfer energy (Δ).^[^ ^27^ ^]^ The term *U* is used to describe the d–d Coulomb, whereas Δ depends on the electronegativity difference. Ti^4+^ can have zero *U* value and large Δ value since Ti^4+^ has no 3d electrons and large electronegativity difference with O, leading to the formation of the *U*≪Δ system and electrochemical inactivity.^[^ ^28^ ^]^ Ti^4+^ does not participate in the redox reaction, but it can produce a lot of Li‐O‐Li configurations that can activate the oxygen redox reaction because it can form easily Li_2_TiO_3_‐like structure and can maintain the cation‐disorder structure, which can provide the structure stability even with large amount of Li extraction.^[^ ^29^ ^]^ Furthermore, given that Ti—O has a strong bonding energy than other transition metal (TM)—O, Ti^4+^ can stabilize O atom in the structure.^[^ ^30^ ^]^

**Figure 5:**
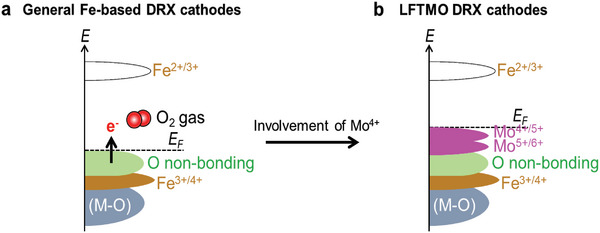
Schematic band structure of a) general Fe‐based DRX cathodes, and b) LFTMO DRX cathodes.

Given that the Fe^3+^ shows *U* > Δ,^[^ ^11^ ^]^ in the typical Fe‐based cation‐disordered system the non‐bonding oxygen state can be destabilized, resulting in the irreversible O redox and O loss in Li‐Fe‐Ti‐O (**Figure** **5**). When Fe redox reaction can be active in typical Fe‐based DRX materials, the irreversible redox reaction can be easily occurred.^[^ ^11^ ^]^ As a result, other redox‐active species are needed for stabilizing the oxygen redox reaction^[^ ^31^ ^]^ and TM redox reaction without using Fe redox reaction via appropriate electronic band alignment. In this study, Mo serves as an active redox center instead of Fe in Fe‐based DRX materials. Introducing Mo^4+/5+^ into Li‐Fe‐Ti‐O material enables Mo^4+^/Mo^6+^ redox reaction instead of Fe redox reaction and stabilizes the oxygen redox reaction resulting from the electronic state of Mo above the non‐bonding O state (*U*/2 ≈ Δ) (Figure 5). Furthermore, as Mo^4+^ is oxidized to Mo^6+^ in the middle of first charge, the Mo ion can be migrated and then the local structure can be distorted after first cycle. This local structure changes in subsequent cycles can stabilize the oxygen redox reaction by broadening the non‐bonding O state, resulting in a reversible oxygen redox reaction. After first cycle, Fe^3+^, Ti^4+^, and Mo^6+^ can also help maintain the disordered structure.

The reduction of Fe^3+^ in typical Fe‐based DRX materials results in low‐voltage Fe^2+^/Fe^3+^ redox reaction, which significantly lowers the average discharge potential (Figure S8a, Supporting Information).^[^ ^10^ ^]^ However, Mo can be reduced to lower oxidation state instead of Fe because of the multivalent Mo state in the density of states (DOS) below the Fe^2+/3+^ state during the discharge process. As a result, the Mo state can play the role of a redox buffer, resulting in high average discharge potential, as illustrated in Figure S8b (Supporting Information).^[^ ^32^ ^]^ As a result, particles in LFTMOs may not be pulverized upon cycling because Fe‐related redox reaction does not occur actively.

Nevertheless, LFTMO did not achieve higher performance compared to other layered cathode materials^[^ ^33^ ^]^ in terms of capacity retention or rate capability (Figures S9–S11, Supporting Information). Especially, although the sample with x = 4 can induce reversible oxygen redox reaction due to the addition of an optimized amount of Mo, it suffers from more severe capacity fading than the other samples. Possible reason for this can be the fact that there is more lithiation and delithiation in the sample caused by the increased oxygen redox activity. In order to overcome these drawbacks, further research for improving rate capability and cycle stability will be needed in the future.

## 4 Conclusion

In summary, we report on novel Fe‐based disordered rocksalt Li_1.2_Fe_1/3_Ti*_x_*_/15_Mo_(7‐_ *_x_*_)/15_O_2_ (LFTMO) materials that operate stably with high capacity and high average discharge potential. We compared the structural and electrochemical properties by controlling the ratio of Ti to Mo in the samples. We found out that the introduction of Mo into the Fe‐based DRX materials helps stabilize the oxygen redox reaction without involving Fe redox reaction and changes the local structure in first charge. This can be partly because Mo redox reaction occurs instead of Fe redox reaction and then Mo^6+^ ions easily changes the local structure that can enable to reversible oxygen redox reaction in first cycle. As a result, the sample with *x* = 4 can achieve ≈238 mAh g^‐1^ discharge capacity even at room temperature with ≈2.7 V of average discharge voltage via optimizing the oxygen redox reaction and Mo redox reaction. Furthermore, particles are almost maintained without severe pulverization that is typically found in Fe‐based DRX materials. The findings to control redox reactions without activating Fe redox reaction via the doping of other redox‐active elements will provide a new scope for the strategy in the design of low‐cost Fe‐based DRX materials that can achieve high energy density with long‐term cycle.

## 5 Experimental Section

### Preparation of Materials

LFTMOs (Li_1.2_Fe_1/3_Ti*_x_*_/15_Mo_(7‐_ *_x_*_)/15_O_2_, 3 ≤ *x* ≤ 6) were synthesized by a solid‐state reaction. To prepare LFTMOs, a stoichiometric amount of Li_2_CO_3_, Fe_2_C_2_O_4_∙2H_2_O, TiO_2_ and MoO_3_ was ball‐milled in acetone for 15 h. To synthesize the LFTMOs, a mix of the precursors was heated for 15 h in Ar at 1050 °C. For a carbon coating, synthesized LFTMOs and 10 wt% citric acid were dissolved in ethanol and mixed using planetary wet ball‐milling (PBM, Fritsch Pulverisette planetary ball‐mill) for 5 h with 500 rpm. A mix of LFTMOs and citric acid was heated for 3 h in Ar at 700 °C.

### Materials Characterization

XRD (Max‐2500, Rigaku) was used to characterize the structures in LFTMOs. XRD measurements were collected from 15° ≤ 2*θ* ≤ 70° at a rate of 3° min^‐1^ using CuK*α* radiation operating at 40 kV and 200 mA. Rietveld refinement was done by using HighScore Plus software. The ex situ synchrotron X‐ray diffraction measurements were performed on beamline 9B‐HRPD at Pohang Accelerator Laboratory (PAL), Pohang, Korea. The incident X‐rays were vertically collimated by a mirror, then monochromated to a wavelength of 1.4970 Å by a double‐crystal Si (111) monochromator. The datasets were collected in the range of 30° ≤ 2*θ* ≤ 140.5° with a step size of 0.01°. Rietveld refinement was done by using Full Proof software. Hard X‐ray absorption spectra of Fe, Ti, and Mo K‐edge were collected at beamline 7D a Pohang Accelerator Laboratory (PAL), Pohang, Korea in transmission mode with the N_2_ gas‐ionization detectors and a Si(111) double‐crystal monochromator detuned ≈70% of its original intensity to eliminate higher‐order harmonics. The storage ring was operated at 2.5 GeV with an injection current of ≈350 mA. The spectral energies were calibrated by using the first inflection points in the Fe, Ti, and Mo metal foil spectra as references.

### Electrochemical Measurement

For electrochemical test, composite electrodes were made by mixing active material (80 wt%), Super‐P carbon (Timcal, 15 wt%), and binder (polyvinylidene fluoride, 5 wt%). A slurry mixture was tape‐cast on Al foil by the doctor‐blade method. The cells were assembled in an Ar‐filled glove box and tested them on a Maccor 2200 operating in galvanostatic mode, using Li metal as an anode, nonaqueous electrolyte (1 m LiPF_6_ in ethylene carbonate: diethyl carbonate (1:1 vol%, PANAX ETEC Co. Ltd., battery grade), and Celgard 2400 as a separator in a 2032‐coin cell. The loading level of all electrodes was about 2 mg cm^‐2^. All cells were tested at room temperature.

## Conflict of Interest

The authors declare no conflict of interest.

## Supporting information

Supporting Information

## Data Availability

The data that support the findings of this study are available from the corresponding author upon reasonable request.
