# Supplementary material for: A New Class of High‐Capacity Fe‐Based Cation‐Disordered Oxide for Li‐Ion Batteries: Li‐Fe‐Ti‐Mo Oxide
Source: Adv Sci (Weinh). 2023 Apr 23;10(18):2300615. doi: 10.1002/advs.202300615 (PMC10288230; doi:10.1002/advs.202300615)
Supplement: Supplementary file 1 — Supporting Information [file ADVS-10-2300615-s001.pdf]

Supporting Information

A new class of high-capacity Fe-based cation-disordered oxide for Li-ion batteries: Li-Fe-Ti-Mo oxide

*Jieun Kim, Yongho Shin, and Byoungwoo Kang**

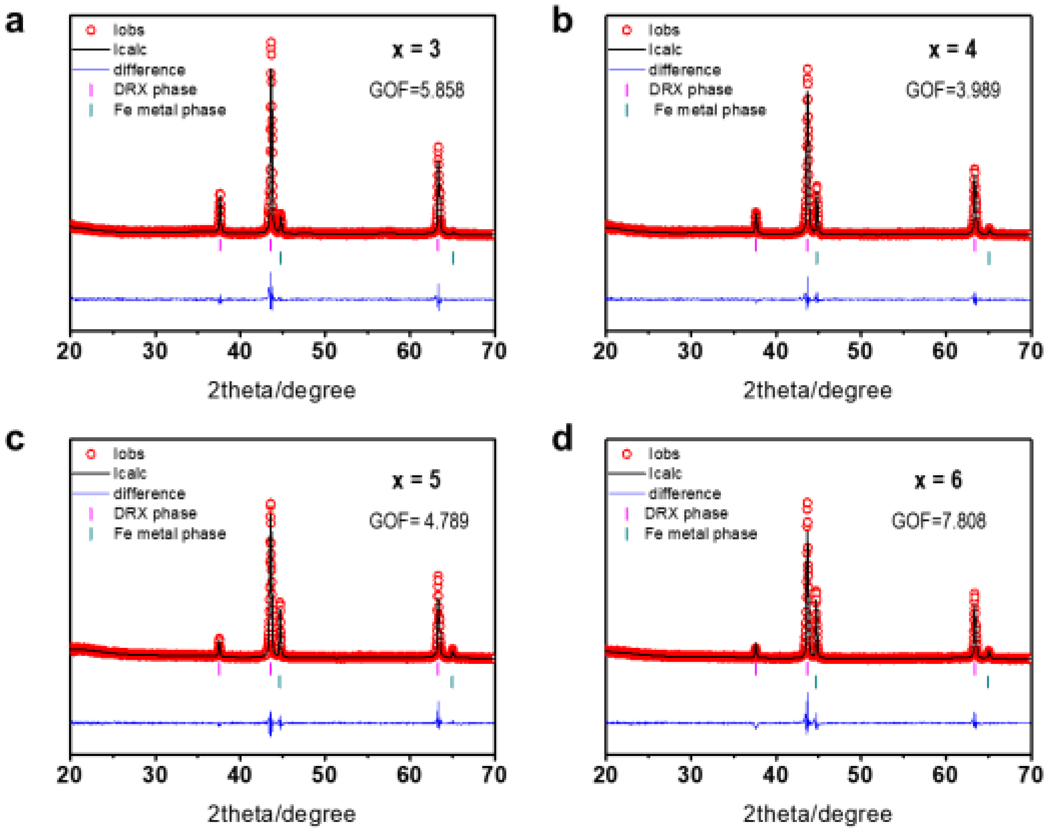

Figure S1. The XRD refinement results of **(a)** x=3, **(b)** x=4, **(c)** x=5, and **(d)** x=6 sample.

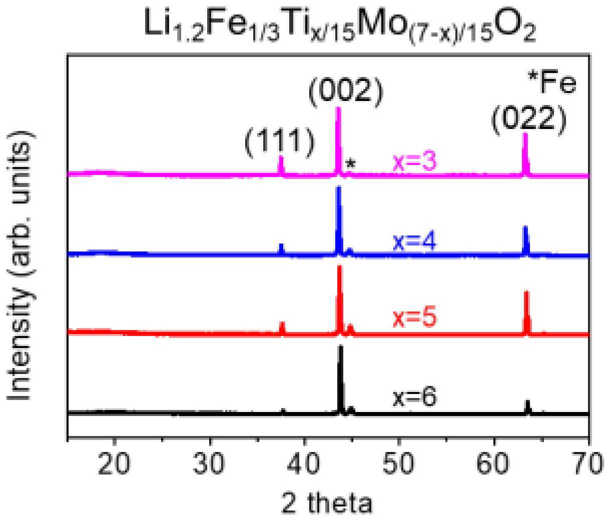

Figure S2. XRD patterns of the synthesized sample.

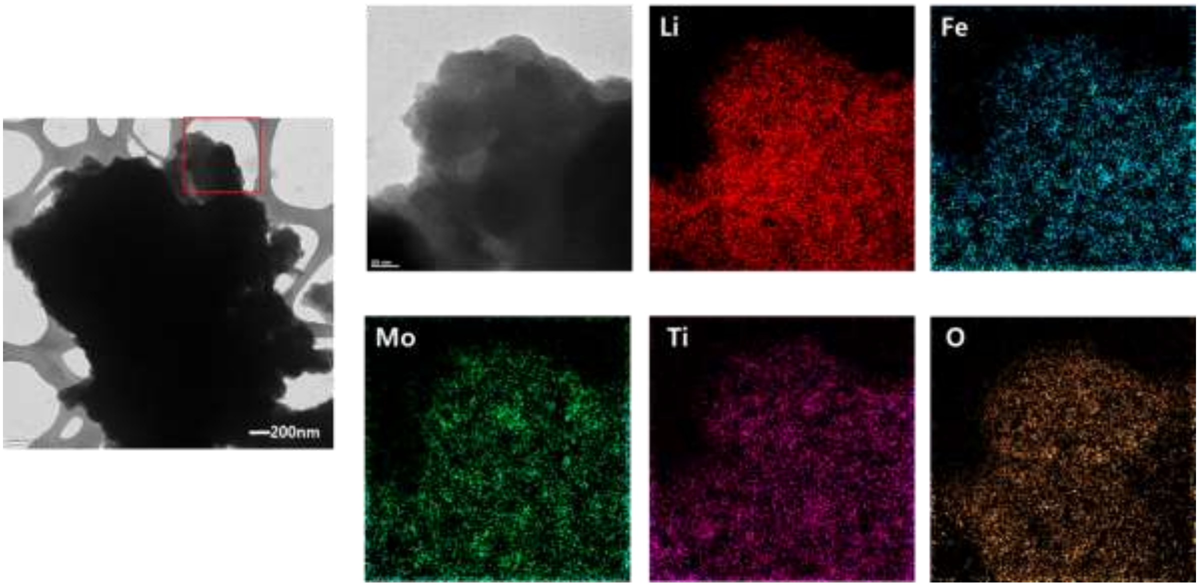

Figure S3. TEM image and EELS elemental mapping of x=5 sample of the region in the red box.

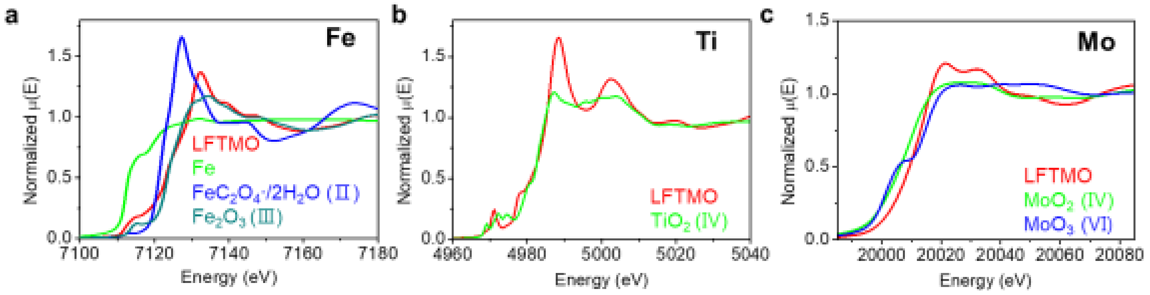

Figure S4. XANES spectra of **(a)** the Fe K-edge, **(b)** Ti K-edge and **(c)** Mo K-edge of pristine x=5 sample.

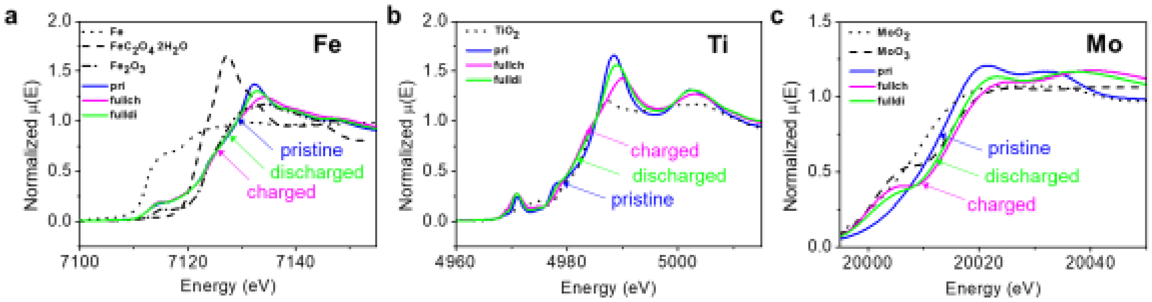

Figure S5. XANES spectra of **(a)** Fe K-edge, **(b)** Ti K-edge, and **(c)** Mo K-edge during initial cycle.

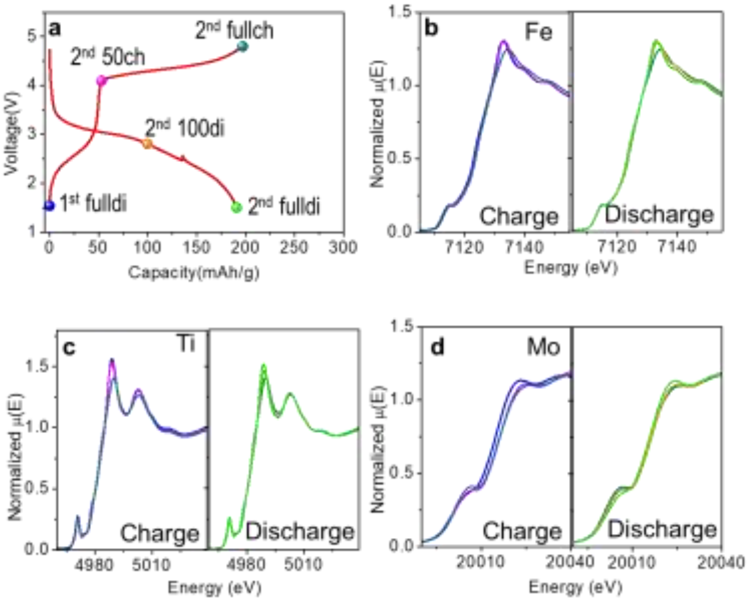

Figure S6. XANES spectra of **(a)** Fe K-edge, **(b)** Ti K-edge, and **(c)** Mo K-edge during 2nd cycle.

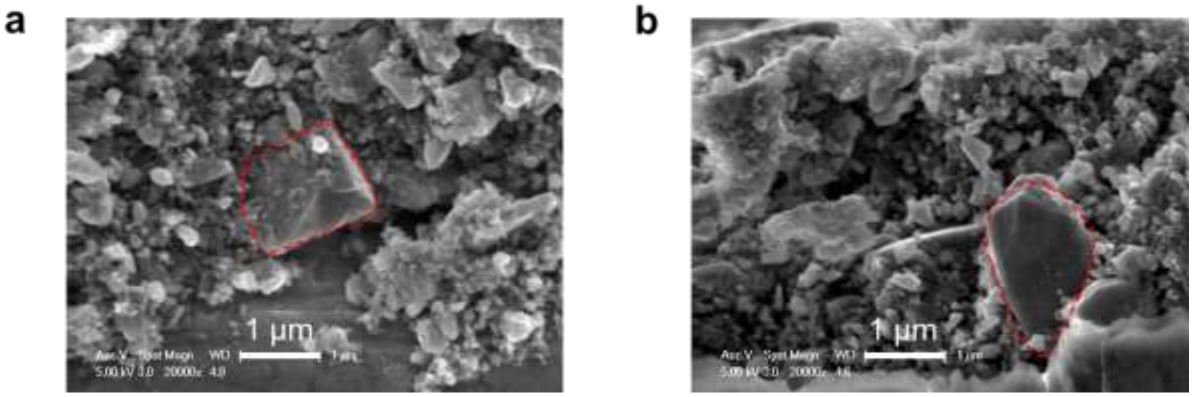

Figure S7. SEM images of the electrode **(a)** before cycles, and **(b)** after 50cycles of x=4 sample.

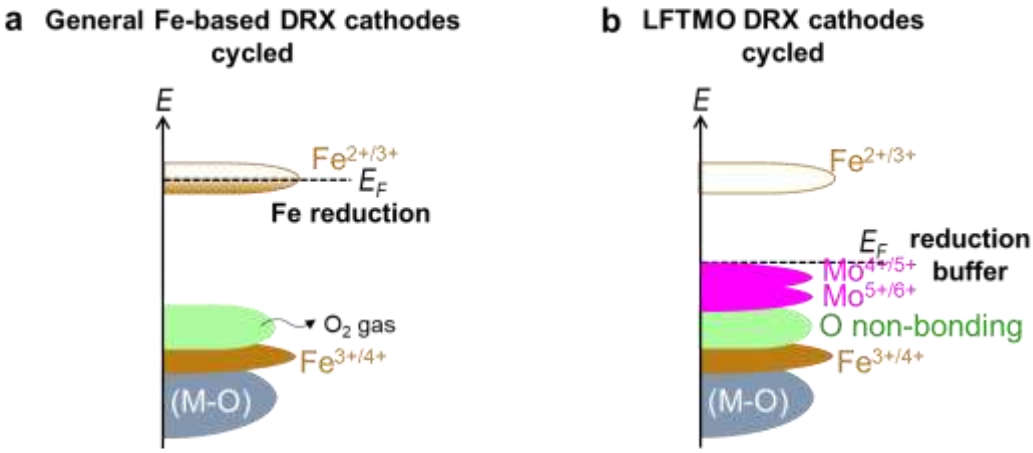

Figure S8. Schematic band structure of **(a)** cycled general Fe-based DRX cathodes, and **(b)** cycled LFTMO DRX cathodes.

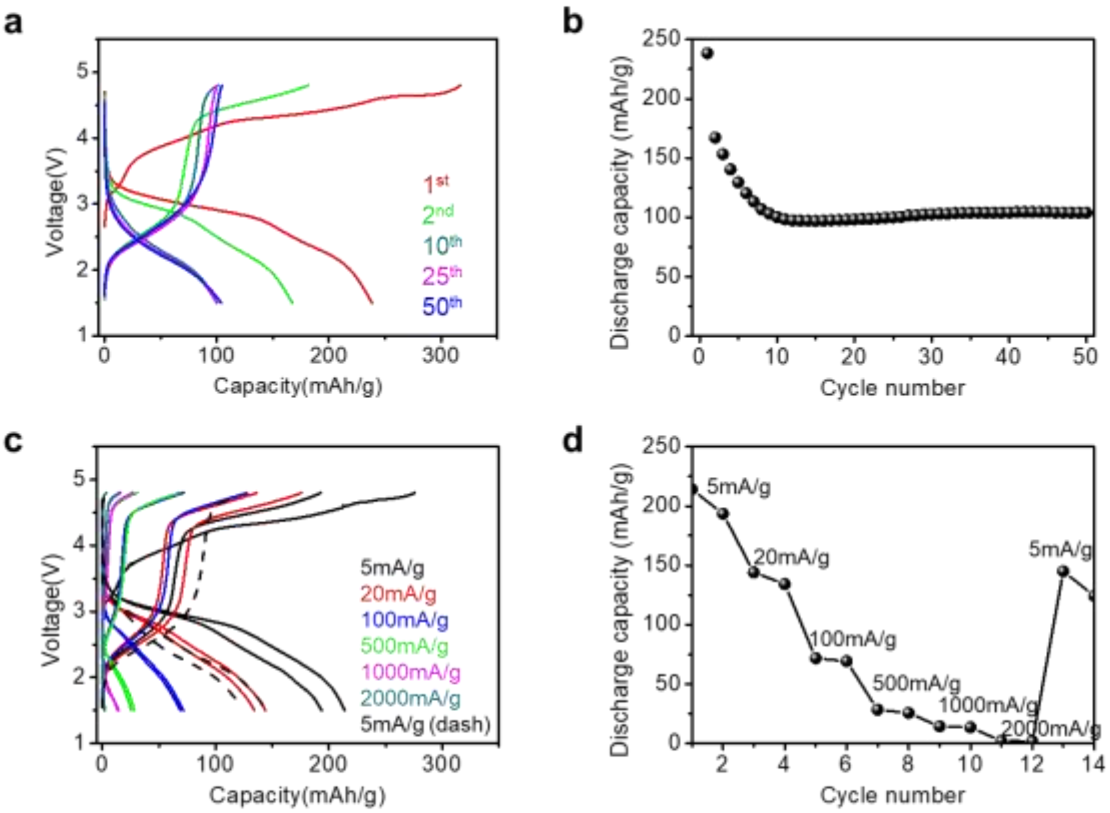

Figure S9. (a) Voltage profiles and **(b)** capacity retention of x=4 sample in the voltage range 1.5V-4.8V at room temperature. The half-cell is cycled at 20mA/g after first cycle at 5mA/g. **(c)** Voltage profiles and **(d)** discharge capacity in discharge rate capability test of x=4 sample with 5mA/g charge rate.

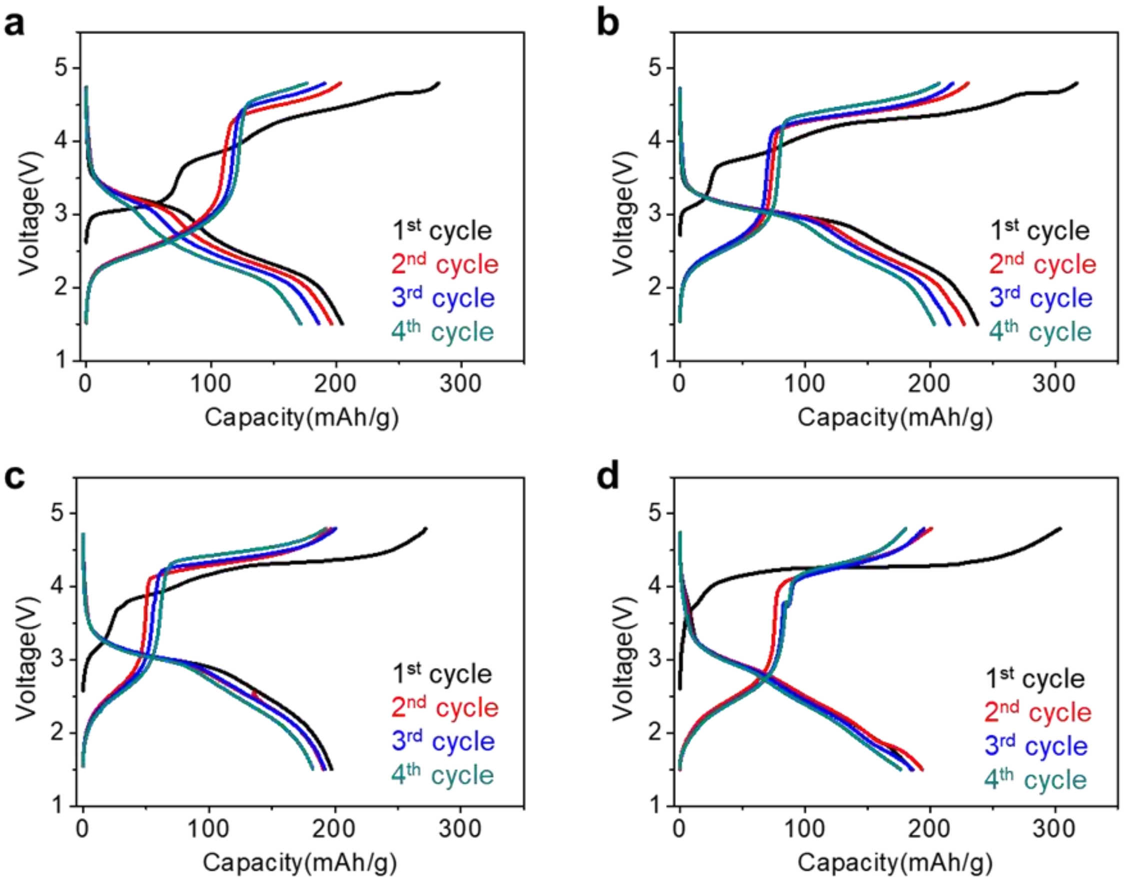

Figure S10. Voltage profiles of **(a)** x = 3, **(b)** x = 4, **(c)** x = 5, and **(d)** x = 6 sample during 4 cycles (Current density = 5 mA·g^{-1}).

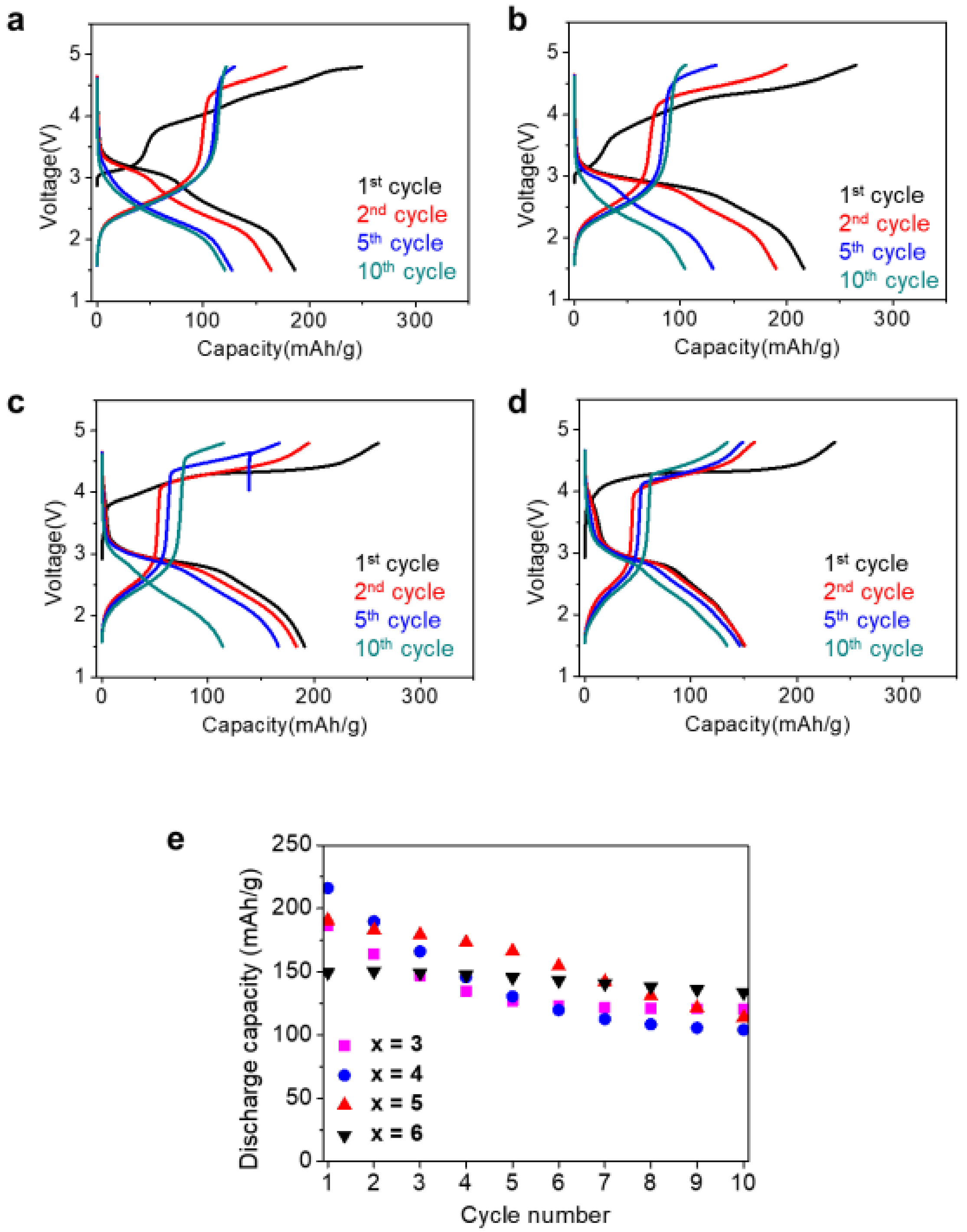

Figure S11. Voltage profiles of **(a)** x = 3, **(b)** x = 4, **(c)** x = 5, and **(d)** x = 6 sample and **(e)** capacity retention during 10 cycles (Current density = 20 $mA \cdot g^{-1}$).

Table S1. Details for LFTMO from XRD refinement

Sample (x)	Lattice parameter *a*(Å)	Fe metal (wt%)
x = 3	4.162	2.5
x = 4	4.157	5.6
x = 5	4.155	5.9
x = 6	4.151	8.4

Table S2. Calculated compositions of LFTMO based on the refinement of XRD results.

Sample	Formula	Li (mol)	Fe (mol)	Ti (mol)	Mo (mol)	O (mol)	Theoretical capacity (total/Mo-based) (mAh/g)
**x=3**	**Li1.2 fix**	1.2	0.292	0.2	0.267	1.958	353.0/156.9
	**O2 fix**	1.226	0.298	0.204	0.272	2	
**x=4**	**Li1.2 fix**	1.2	0.244	0.267	0.2	1.910	380.8/126.9
	**O2 fix**	1.256	0.255	0.279	0.209	2	
**x=5**	**Li1.2 fix**	1.2	0.242	0.333	0.133	1.909	396.3/88.1
	**O2 fix**	1.257	0.254	0.349	0.140	2	
**x=6**	**Li1.2 fix**	1.2	0.209	0.4	0.067	1.876	425.6/47.3
	**O2 fix**	1.279	0.223	0.426	0.071	2	

Table S3. Summarized electrochemical behavior of LFTMO

Sample (x)	Charge capacity (mAh/g)	Discharge capacity (mAh/g)	Coulombic efficiency (%)
x = 3	281.4	204.5	72.7
x = 4	317.2	237.9	75.0
x = 5	272.2	197.4	72.5
x = 6	303.9	185.1	60.9

Table S4. Summary of reported Fe-based DRX materials and LFTMO (x=4) (This work) in terms of their electrochemical performance, and condition.

Cathode	Electrochemical performance		Electrochemical condition	
	Discharge capacity at 1st cycle	Coulombic efficiency at 1st cycle	Current density	Voltage range
**This work (LFTMO (x=4 sample))**	**237.9 mAh/g**	**75.0%**	**5 mA/g**	**1.5-4.8V**
$Li_{1.18}Fe_{0.34}Ti_{0.45}O_2$[1]	223.4 mAh/g	84.5%	10 mA/g	**1.5-4.8V**
$Li_{1.2}Fe_{0.4}Ti_{0.4}O_2$[2]	153 mAh/g	57.5 %	7.5 mA/g	2.5-4.8V
Li_2FeTiO_4/graphene[3]	218.8 mAh/g	76.2 %	10 mA/g	1.5-5.0V
$LiFeO_2$[4]	214.2 mAh/g	131.8 %	28.2 mA/g	1.5-4.5V
$Li_{1.3}Fe_{0.4}Nb_{0.3}O_2$[5]	225 mAh/g	63.2 %	20 mA/g	1.5-4.8V

Table S5. ICP results of x=5 sample.

Materials	Li	Fe	Ti	Mo
Target atomic ratio	1.2	0.333	0.333	0.133
Measured atomic ratio	1.2	0.329	0.337	0.139

References

[1] M. Yang, J. Jin, Y. Shen, S. Sun, X. Zhao, X. Shen, *ACS Applied Materials & Interfaces* **2019**, *11* (47), 44144.
[2] M. Tabuchi, A. Nakashima, H. Shigemura, K. Ado, H. Kobayashi, H. Sakaebe, K. Tatsumi, H. Kageyama, T. Nakamura, R. Kanno, *J Mater Chem* **2003**, *13* (7), 1747.
[3] M. Yang, X. Zhao, C. Yao, Y. Kong, L. Ma, X. Shen, *Materials Technology* **2016**, *31* (9), 537.
[4] Z.-j. Zhang, J.-Z. Wang, S.-L. Chou, H.-K. Liu, K. Ozawa, H.-j. Li, *Electrochim Acta* **2013**, *108*, 820.
[5] Z. Lebens-Higgins, H. Chung, I. Temprano, M. Zuba, J. Wu, J. Rana, C. Mejia, M. A. Jones, L. Wang, C. P. Grey, Y. Du, W. Yang, Y. S. Meng, L. F. J. Piper, *Batteries & Supercaps* **2021**, *4* (5), 771.
